# Effectiveness of permissive weight bearing in surgically treated trauma patients with peri- and intra-articular fractures of the lower extremities: a prospective comparative multicenter cohort study

**DOI:** 10.1007/s00590-023-03806-5

**Published:** 2023-12-30

**Authors:** Pishtiwan Kalmet, Cherelle Maduro, Coen Verstappen, Guido Meys, Yvette van Horn, Raoul van Vugt, Heinrich Janzing, Alexander van der Veen, Coen Jaspars, Jan Bernard Sintenie, Taco Blokhuis, Silvia Evers, Henk Seelen, Peter Brink, Martijn Poeze

**Affiliations:** 1 Maastricht University Medical Center+, Maastricht, The Netherlands; 2 Adelante Rehabilitation Center, Hoensbroek, The Netherlands; 3 Zuyderland Medical Center, Heerlen, The Netherlands; 4 Viecuri Medical Center, Venlo, The Netherlands; 5 Catharina Hospital, Eindhoven, The Netherlands; 6 Maxima Medical Center, Veldhoven, The Netherlands; 7 Elkerliek Hospital, Helmond, The Netherlands; 8 Maastricht University, Care and Public Health Research Institute (CAPHRI), Maastricht, The Netherlands; 9 Trimbos Institute, Netherlands Institute of Mental Health and Addiction, Utrecht, The Netherlands; 10 Nutrim School for Nutrition, Toxicology and Metabolism, Maastricht University, Maastricht, The Netherlands

**Keywords:** Permissive weight bearing, Restricted weight bearing, Trauma patients, Fractures, Lower extremity, Rehabilitation

## Abstract

**Purpose:**

The aim of the present study was to investigate the effectiveness of a novel approach involving permissive weight bearing (PWB) in surgically treated trauma patients with peri- and intra-articular fractures of the lower extremities.

**Methods:**

Prospective comparative multicenter cohort study in one level 1 trauma center and five level 2 trauma centers. Surgically treated trauma patients with peri- and intra-articular fractures of the lower extremities were included. Permissive weight bearing (PWB) in comparison to restricted weight bearing (RWB) was assessed over a 26-week post-surgery follow-up period. Patients’ self-perceived outcome levels regarding activities of daily living (ADL), quality of life (QoL), pain and weight bearing compliance were used.

**Results:**

This study included 106 trauma patients (N = 53 in both the PWB and RWB groups). Significantly better ADL and QoL were found in the PWB group compared to the RWB group at 2-, 6-, 12- and 26-weeks post-surgery. There were no significant differences in postoperative complication rates between the PWB and RWB groups.

**Conclusion:**

PWB is effective and is associated with a significantly reduced time to full weight bearing, and a significantly better outcome regarding ADL and QoL compared to patients who followed RWB regimen. Moreover, no significant differences in complication rates were found between the PWB and RWB groups.

**Level of evidence:**

Level II.

**Registration:**

This study is registered in the Dutch Trial Register (NTR6077). Date of registration: 01-09-2016.

## Introduction

The recommendations for aftercare in surgically treated trauma patients with peri- and intra-articular fractures of the lower extremities are still more or less the same as they were during the last 60 years, without any source of evidence being given for the advice of restricted weight bearing [1]. In view of this lack of evidence, many orthopedic and trauma surgeons tend to advise conservatively regarding postoperative management and hold on to the prevailing dogmas, i.e., non-weight bearing or restricted weight bearing [2].

The current recommendations regarding postoperative management in surgically treated trauma patients with peri- and intra-articular fractures of the lower extremities is either non-weight bearing or restricted weight bearing for 6–12 weeks, followed by partial weight bearing with a 25% increase in weight every week [1, 3].

There is no consensus from the surgeons in the current postoperative management [4]. Moreover, almost 90% of the surgeons deviate from the current postoperative management protocols because of e.g., type of fracture, (un-) certainty of fixation, clinical experience, or gut feeling [4]. Furthermore, while instructions on rehabilitation provided to patients may be clear, patients’ compliance with a non-weight bearing or restricted weight bearing regimen is poor, so neither surgeons nor patients follow the instructions regarding the postoperative management regimen [5, 6].

The postoperative management of surgically treated peri- and intra-articular fractures of the lower extremities is very important in view of the impact on the patient’s functional outcome. Recent literature has reported composite postoperative complication rates of up to 37%, with an average of 10–20% for patients with lower extremity fractures [7–11]. In addition, several studies indicate that the postoperative management, i.e., early or permissive weight bearing, increases the postoperative complications rates [3, 4].

Several biomechanical, animal, and clinical studies have found early or permissive weight bearing to be beneficial [2, 3, 12–15]. However, very few clinical studies are available that compared permissive weight bearing (PWB) with restricted weight bearing (RWB) in surgically treated trauma patients with peri- and intra-articular fractures of the lower extremities. Furthermore, despite the generally accepted value of the use of patient-specific outcome measures, no data is available offering insights into patients’ self-perceived outcome levels (e.g., regarding activities of daily living (ADL), quality of life or pain) in PWB versus RWB.

The aim of the present study was therefore to investigate the effectiveness of PWB in surgically treated trauma patients with peri- and intra-articular fractures of the lower extremities, reporting on patients’ self-perceived outcome levels regarding ADL, quality of life, pain, weight bearing or patients’ compliance and postoperative complications, in comparison to RWB, over a 26-week post-surgery follow-up period.

## Materials and methods

### Study design and participants

This prospective comparative multicenter cohort study included surgically treated trauma patients with peri- and intra-articular fractures of the lower extremities. Subjects were consecutively recruited from six hospitals in the Netherlands between October 2017 and September 2018. The allocation of the patients to the intervention or control group depended on the regimen adhered to by the hospital in which the patients were surgically treated. During the conceptualization of this study design, randomization was not considered feasible because of the nature of the two different interventions. Namely, implementation of these different protocols includes patient instructions as well as physical therapy guidance and nursing staff participation. A mix of treatment protocols on a single ward was therefore considered suboptimal. However, this meant we had to consider that not randomizing the study could introduce observer bias, which may be a study limitation. Patients from two hospitals underwent the PWB protocol as aftercare rehabilitation treatment, while the others followed the RWB protocol [1, 2].

Surgically treated trauma patients with peri- and intra-articular fractures of the lower extremities (i.e., pelvic fractures, acetabular fractures, distal femur fractures, tibial plateau fractures, pilon fractures, calcaneal fractures and talar fractures) were eligible for inclusion if they were aged 18 years or over. Patients with pathological fractures, shaft fractures treated with intra-medullary nailing, hip fractures treated with prosthesis, or fractures treated with external fixation, and patients with amputations in the area of the lower extremity, were excluded. Patients with inability to follow instructions (e.g., cognitive dysfunction), due to the consequences of severe neurotrauma or due to concomitant or mental illness were also excluded [16].

### Protocols

The PWB treatment involves a gradual progression in functional activities guided by patients’ subjective experience (pain and confidence to bear weight) and by objective clinical symptoms of the patients occurring during the process of rehabilitation, evaluated by the physical therapist during every outpatient physiotherapy session. Clinical symptoms include the evolution of signs of inflammation, neuro-vascular status, weight-bearing tolerance, changes in the alignment of the affected side of the body, and the quality and function of the soft tissue and the joints involved. The progress in therapy is not determined by any predetermined or fixed degree of loading of the affected side in kg or in percentage of bodyweight, as this has proved to be difficult to adhere to. This process enables patients to carry out the activities with normal/optimal motor skills as soon as possible. The approach is guided by the quality of performance and the safety of the activity (e.g., preventing stumbling). The next stage of the treatment is started when the gait pattern associated with the current stage of the treatment is optimally executed and can be performed by the patient safely and independently [2].

In the RWB group, the patients underwent a non-weight bearing regimen for 6–12 weeks followed by partial weight bearing with a 25% increase in weight loading every week according to the existing (AO) guidelines [1].

### Outcome measures and co-variables

The patients’ self-perceived outcome levels, questionnaires related to the activities of daily living (ADL) were assessed as primary outcome measure. ADL was measured with the Lower Extremity Functional Scale (LEFS). The LEFS consists of 20 questions about a person’s ability to perform daily tasks. The score for each question ranges from 0 (extreme difficulty in performing the activity) to 4 (good performance of activity), maximizing the score at 80 points. The lower the score, the greater the disability [17].

The other patients’ self-perceived outcome levels were assessed as secondary outcome measures, using questionnaires related to the quality of life and pain score. The quality of life was measured with the Short Form-12 (SF-12) questionnaire. The SF-12 consists of 12 items that assess 8 dimensions of health: physical functioning, role-physical, bodily pain, general health, vitality, social functioning, role-emotional and mental health. The SF-12 measures various aspects of physical and mental health from which a physical composite score (PCS) and a mental composite score (MCS) can be calculated, ranging from 0 to 100 [18]. The intensity of pain was measured with the Numeric Rating Scale (NRS; 0 indicating no pain and 10 worst pain) [19]. All patients’ self-perceived outcome levels were obtained at the follow-up time-points of 2-, 6-, 12- and 26-weeks post-surgery. The other secondary outcome measures were the rehabilitation outcome (i.e., outpatient physiotherapy, time to full weight bearing, completion of rehabilitation within 26 weeks), complications during a 26-week post-surgery follow-up and the progression of weight bearing during the first 12 weeks of rehabilitation. Postoperative complications, i.e., superficial wound infections, deep wound infections, non-unions and secondary dislocations, or other additional adverse situations that required medical intervention, were recorded as either present or non-present, along with the type of complication. Removal of implants was only performed in case of functional complaints.

All patients’ compliance were monitored for 3 months after surgery with the OpenGo insole (Moticon GmbH, Munich, Germany) [20]. The insole incorporates 13 capacitive pressure sensors and a 3D accelerometer, measuring peak pressures (in Newton) and mean weight bearing (in Newton). It operates completely wireless. Data is stored on a flash drive. The insole can be placed in any shoe and shoes can be changed at random during the study due to an automated zeroing system [20].

Baseline characteristics, recorded at admission, included age at time of fracture, sex, ASA (American Society of Anesthesiologists) classification (type 1–6) [21], Charlson comorbidity score (classifying prognostic comorbidity, a higher score correlating with additional comorbidities) [22], type of fracture and in-hospital length of stay (in days).

## Statistical analysis

Statistical analysis was performed with IBM SPSS Statistics (Version 25.0, Armonk, NY). Descriptive statistics were used to describe the demographic data and baseline characteristics for the entire study population. Independent samples t-tests were used for normally distributed continuous data, and chi-squared tests for categorical variables. In the case of non-parametric data, the median with the interquartile range was calculated. Furthermore, a linear mixed model was used to identify any differences among the outcome measures over time. This analysis ensured that both random and cluster effects, such as treatment in different hospitals, and fixed effects, such as ASA classification, could be considered and corrected for. Results are presented as either mean (standard deviation) or frequencies and percentages. The level of statistical significance was set at α = 0.05. The data was analyzed blinded by the researchers.

## Results

### Baseline characteristics

**Fig. 1:**
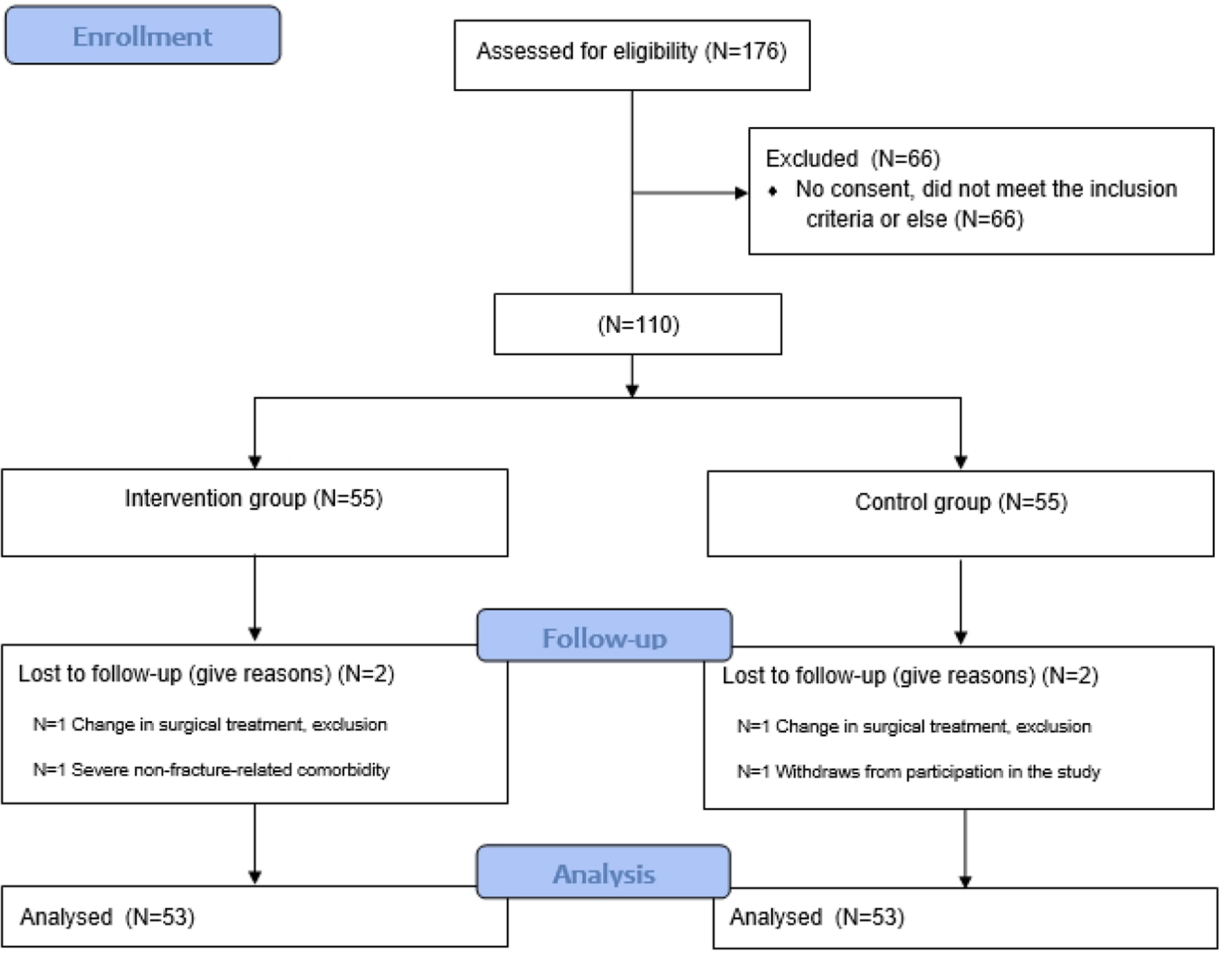
CONSORT Flowchart of study patients

A total of 106 patients were included in this cohort study, N = 53 in both the PWB and RWB groups (Fig. 1). As the assumption for normality was violated, non-parametric tests were used, and established that the PWB group patients had comparable ASA score (p = 0.14) and fewer comorbidities, as measured with the Charlson score, (p = 0.03) compared to those in the RWB group. No significant differences in sex, age, type of fracture, number of surgical interventions during primary admission or in-hospital length of stay were found between the groups. Characteristics of patients in the PWB and RWB groups are summarized in Table 1.

**Table 1:** Baseline characteristics and in-hospital outcome of the PWB and RWB groups

	**PWB** (N = 53)	**RWB** (N = 53)	**Total** (N = 106)	p
Female, N	27 (50.9%)	27 (50.9%)	54 (50.9%)	1.00
Median age (IQR), years	55.0 (38.5–65.0)	60.0 (47.0–67.0)	58.0 (43.5–66.3)	0.27
ASA, N I, II > II	49 (92.5%) 4 (7.5%)	44 (83.0%) 9 (17%)	93 (87.7%) 13 (12.3%)	0.14
Median Charlson score (IQR)	1 (0–3)	2 (1–3)	2 (0–3)	0.03
Fracture type, N: Pelvic Acetabular Tibial plateau Pilon Calcaneal	7 (13.2%) 5 (9.4%) 16 (30.2%) 17 (32.1%) 8 (15.1%)	1 (1.9%) 3 (5.7%) 28 (52.8%) 12 (22.6%) 9 (17.0%)	8 (7.5%) 8 (7.5%) 44 (41.5%) 29 (27.4%) 17 (16%)	0.18
**In-hospital outcome:** Two or more procedures (%) Median length of stay (IQR), in days	9 (17.0) 7.0 (2.0–15.5)	8 (15.1) 5.0 (2.0–11.5)	17 (16.0) 6.0 (2.0–14.0)	0.57 0.24

Abbreviations: *PWB* permissive weight bearing, *RWB* restricted weight bearing,* N* number of subjects, *ASA* American Society of Anesthesiologists, *IQR* interquartile range

### Primary outcome measures

**Fig. 2:**
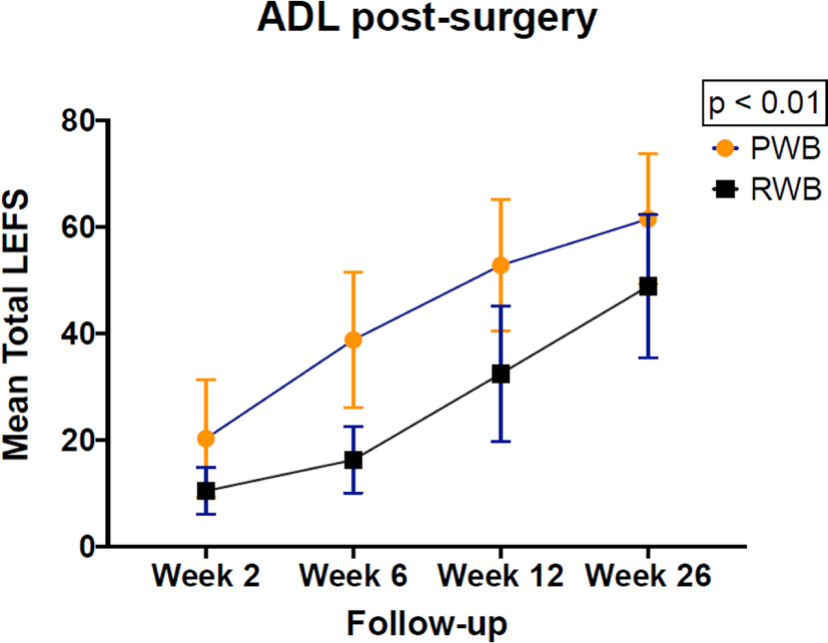
Patient self-perceived activities of daily living during a 26-week post-surgery follow-up. ADL Activities of Daily Living, LEFS Lower Extremity Functional Scale, PWB permissive weight bearing, RWB restricted weight bearing

**Fig. 3:**
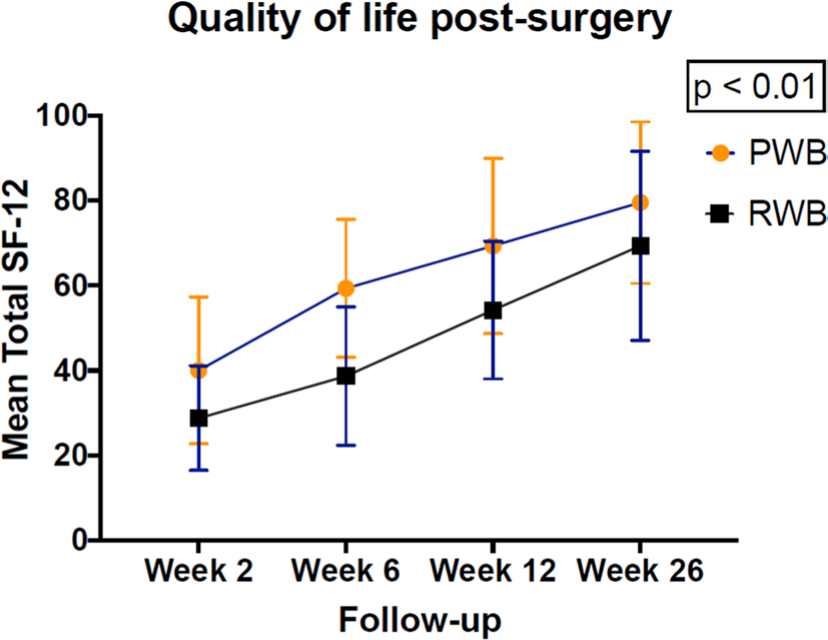
Patient self-perceived quality of life during a 26-week post-surgery follow-up. PWB permissive weight bearing, RWB restricted weight bearing

After a 26 wees post-surgery follow-up, the overall response rate for the patient-specific outcome measures at all measurement points was 99.8% (N = 1 patient refused to fill out the patient self-perceived outcome questionnaires at week 26). ADL as measured with the LEFS, and quality of life as measured with the SF-12, were both significantly better in the PWB group compared to the RWB group (p < 0.01) (Appendix I). There were no differences in pain score between the PWB and RWB groups (Appendix I). The patient self-perceived outcome levels regarding ADL and quality of life in the PWB and RWB groups are summarized in Figs. 2 and 3.

### Rehabilitation and postoperative outcome

Of the total patient population, 77.4% (N = 82) achieved full weight bearing within 12 weeks. The number of patients who achieved this was significantly higher in the PWB group than in the RWB group: 98.1% versus 56.6% (p < 0.01). The median time from surgery to ascertainment of full weight bearing was significantly shorter in the PWB group than in the RWB group: 4.0 (2.1) weeks versus 12.2 (4.2) weeks (p < 0.01). The incidence of postoperative complications in the total study population was 16.0%, with no **statistical** significant differences between the PWB group and the RWB group (11.3% [N = 6] versus 20.8% [N = 11], respectively (p = 0.19), see Table 2. **Among all documented complications, superficial wound infections did not require surgical intervention. Consequently, N = 3 patients adhering to the PWB regimen required supplementary surgical procedures, in contrast to N = 5 patients who adhered to the RWB regimen.**

**Table 2:** Rehabilitation outcome and postoperative complications in the PWB and RWB groups

	**PWB** (N = 53)	**RWB** (N = 53)	**Total** (N = 106)	p
**Prescribed rehabilitation aftercare (%):** PWB 6 weeks RWB 8 weeks RWB 12 weeks RWB **Rehabilitation outcome:** Median OPD (IQR), in hours FWB within 12 weeks (%), N Median time to FWB (IQR), in weeks N who completed rehabilitation within 26 weeks (%)	53 (100) 0 (0) 0 (0) 0 (0) 25 (13.0–46.8) 52 (98.1) 4.0 (2.0–7.0) 30 (65.2)	0 (0) 36 (67.9) 3 (5.7) 14 (26.4) 41 (28.5–57.5) 30 (56.6) 13.0(9.0–15.0) 16 (34.8)	53 (50) 36 (34.0) 3 (2.8) 14 (13.2) 33 (18.5–52.0) 82 (77.4) 8.0 (4.0–13.0) 46 (43.4)	– 0.01 < 0.01 < 0.01 < 0.01
**Total post-operative complications (%)** Non-unions Secondary dislocations Superficial wound infections Deep wound infections Removal of osteosynthesis material	6 (11.3) 0 (0.0) 1 (1.9) 3 (5.7) 1 (1.9) 1 (1.9)	11 (20.8) 1 (1.9) 2 (3.8) 6 (11.3) 1 (1..9) 1 (1.9)	17 (16.0) 1 (0.9) 3 (2.8) 9 (8.5) 2 (1.9) 2 (1.9)	0.19

Abbreviations: *PWB* permissive weight bearing, *RWB* restricted weight bearing, *N* number of subjects, *IQR* interquartile range, *OPD* outpatient physiotherapy duration, *FWB* full weight bearing

### Weight bearing compliance

**Fig. 4:**
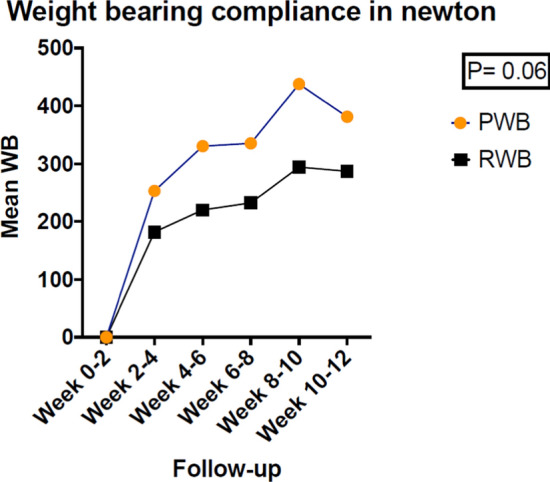
Mean weight bearing compliance during a 26-week post-surgery follow-up. Abbreviation: PWB Permissive weight bearing, RWB Restricted weight bearing, N number of subjects, SD standard deviation, WB Weight bearing in newton on affected leg

**Fig. 5:**
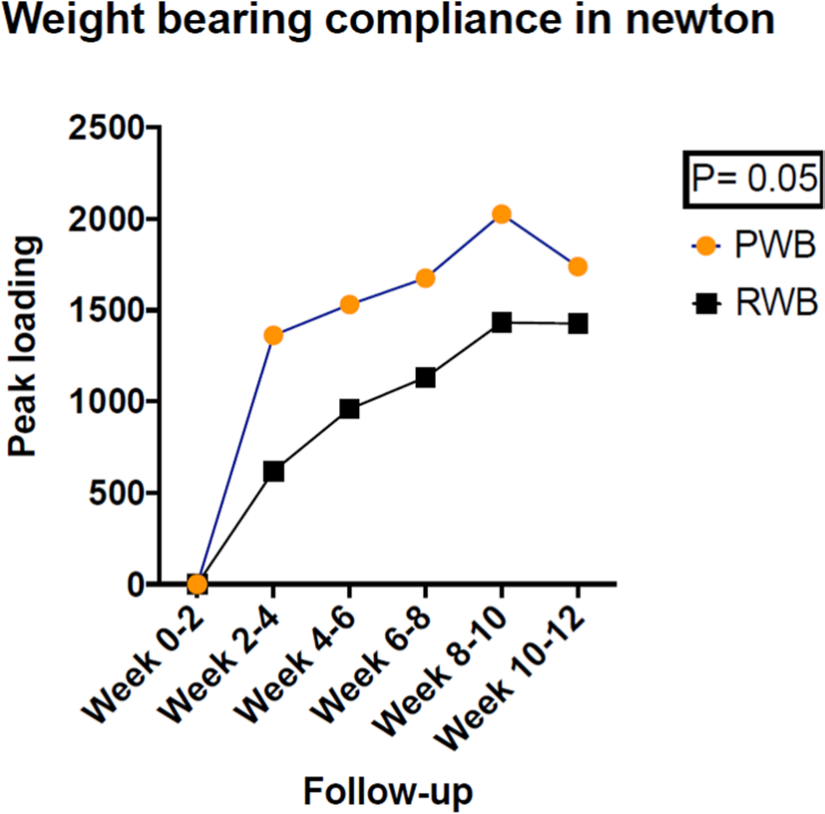
Weight bearing compliance expressed in peak loading during a 26-week post-surgery follow-up. Abbreviations: PWB Permissive weight bearing, RWB Restricted weight bearing, N number of subjects, SD standard deviation, Peak loading Peak loading in newton on affected leg

The mean weight bearing and peak loading were not significantly different between the subjects who followed the PWB or RWB regimens, see Figs. 4 and 5.

## Discussion

The aim of this study was to investigate the effectiveness of a novel approach involving permissive weight bearing (PWB) in surgically treated trauma patients with peri- and intra-articular fractures of the lower extremities. The PWB regimen led to the patients being able to bear full weight on their affected leg much sooner, with a better ADL and quality of life, compared to those who followed the usual RWB regimen. No significant differences between the two treatment regimens were found in either postoperative complication rates or pain levels.

Patients’ self-perceived outcome levels were significantly better among patients who followed the PWB protocol than among those who followed the RWB protocol. This study found a general improvement in ADL (LEFS) and quality of life (SF-12) for both groups during the 26-week rehabilitation period. In our total population, the mean LEFS 26 weeks post-surgery was 55.2 (14.3). This is in line with earlier studies, which found similar levels of ADL in trauma patients after surgery of the lower extremities [23–25]. The mean quality of life for the total population in our study was also in the same range as that reported by other studies [26, 27].

Despite the early PWB regimen, the recorded pain levels during the 26-week rehabilitation period were higher in the RWB group than in the PWB group, which could be due to the consequences of immobilization [2].

In our study 56.6% of the patients in the RWB group were already bearing full weight within 12 weeks, in contrast to the standard protocol of 12 weeks non-weight-bearing or partial weight bearing [1]. Earlier studies also reported that one-third of patients (due to e.g., cognitive impairment in older patients to follow instructions) did not comply with a non-weight-bearing or restricted weight bearing regime [5, 6]. Despite the willingness to comply, patients often do not follow the weight-bearing restrictions and increase their weight bearing as fracture healing progresses [6]. This is also in line with our data on weight bearing, as measured with the Moticon insoles. These measurements showed that there was no significant difference in mean weight bearing between the RWB and PWB groups. The difference in peak loading was nearly significant between the RWB group and PWB group: p = 0.05. The patients in the PWB group were bearing full weight 9 weeks earlier than those in the RWB group. The effort to bear weight earlier was not at the expense of longer duration of outpatient physiotherapy. In fact, the RWB group required significantly longer outpatient physiotherapy than the PWB group, viz. 41 versus 25 h, respectively. Furthermore, significantly more patients in the PWB group completed the rehabilitation within 26 weeks compared to the RWB group, viz. 65.2% versus 34.8%.

Our study found that there was no **statistical** significant difference in postoperative complications between the PWB group and the RWB group. **Nevertheless, a discernible clinical difference was observed, with a lower incidence of post-operative complications evident in patients subjected to the PWB regimen in comparison to those undergoing a RWB regimen.** One of the key objections often raised against early weight bearing is the possibility of fracture displacement [28]. On the other hand, it has often been stated that early weight bearing does not entail an undue risk of postoperative complications [2, 3, 12, 13, 29]. These two views are contradictory, and our study provides evidence in favor of regimes with early weight bearing instead of the standard non-weight-bearing protocols. According to recent literature, a composite postoperative complications rate of up to 27% has been found in surgically treated trauma patients with peri- and intra-articular fractures of the lower extremities [7–11]. Comparison of our complication rates with data published in recent literature shows that we found lower rates of postoperative complication in these patients when they were treated with the PWB regimen. 

Over- and under-loading may lead to prolonged and complicated recovery [2]. A certain minimum level of loading is required to elicit micro-movements between adjacent bony fracture components, stimulating biological processes that are converted into cellular signals initiating bone remodeling [27, 30]. This process is described in the literature as the mechanotransduction in bone. Mechanotransduction is continuously present and enables the bone to resist the mechanical impacts caused by daily activities [30]. To optimize recovery with the lowest number of complications and better patients’ self-perceived outcome levels, one should apply a treatment that approaches the upper limit of the therapeutic bandwidth regarding weight bearing, yet is safe enough to avoid complications due to overloading. This is the case with the PWB protocol [2].

Our study, the first large-scale prospective multicenter cohort study comparing PWB with RWB, adds evidence in support of the use of PWB in surgically treated trauma patients with peri- and intra-articular fractures of the lower extremities. This means that our study contests the paradigm of the current RWB guidelines, which have remained unchanged for 60 years. The time has now come to renew the current guidelines in accordance with the most recent evidence.

When interpreting our data, some limitations have to be considered. Due to practical reasons, this study featured a non-randomized groups design. However, patients were included to the PWB and RWB groups consecutively to avoid selection bias. There were differences regarding the patients’ comorbidities and the different hospitals in which the patients were treated. Our statistical analyses took these issues into consideration, thus correcting the presented results for the confounding influence that these factors may have had on the study results. Surgeon-oriented functional outcome scores (e.g., the function of a knee or ankle joint) were not taken into account. Another limitation of the study is that we did not investigate the cost-effectiveness of PWB. **Therefore, further data are needed on the cost-effectiveness, and long-term patient-reported outcome of the PWB strategy.**

## Conclusion

This prospective, comparative, multicenter study shows that PWB in surgically treated trauma patients with peri- and intra-articular fractures of the lower extremities is effective and is associated with a significant reduction in time to full weight bearing and significantly better outcomes in terms of ADL and quality of life compared to the RWB regime, with a similar complication rate. This PWB protocol contests the paradigm of the current RWB guidelines, which have remained unchanged for 60 years.

## Data Availability

The datasets used and/or analyzed during the current study are available from the corresponding author on reasonable request.
